# Weekday-specific associations of movement behaviors with body composition in preschool children: A compositional data analysis

**DOI:** 10.5114/biolsport.2026.159563

**Published:** 2026-04-20

**Authors:** Jin Guo, Xiangli Liu, Haowen Zhang, Chaonan Wang, Fang Wang, Wei Chen, Di Heng, Qingfeng Dong, Yihua Bao, Zhaoxu Lu, Ting Zhang

**Affiliations:** 1 Capital Center for Children’s health, Capital Medical University, Capital Institute of Pediatrics, Chaoyang District, Beijing, China; 2 Chunjiang Kindergarten, Dongcheng District, Beijing, China; 3 Zhuhai NO.5 Middle School, Zhuhai, China; 4 Global Public Health Center of the Chinese Center for Disease Control and Prevention, Beijing, China; 5 Shougang Preschool Education Center, Beijing, China

**Keywords:** Physical activity, Body composition, Preschool children, Weekdays, Isotemporal substitution

## Abstract

This study aimed to examine the differences in associations between 24-hour movement behaviors and body composition on weekdays versus weekends in preschool children, and to estimate the potential effects of time reallocation among behaviors on body composition. A cross-sectional study was conducted among healthy children aged 3–6 years recruited from urban and suburban kindergartens in Beijing. Movement behaviors were measured objectively using accelerometers on both weekdays and weekends, ensuring comparable assessment across contexts, sleep duration was obtained from parent-completed questionnaires. Body composition was assessed using bioelectrical impedance analysis. Compositional data analysis was applied to build multivariate regression models examining weekday and weekend movement behaviors independently. Isotemporal substitution analysis was used to predict changes in body composition under different time-reallocation among behaviors. Moderate-to-vigorous physical activity (MVPA) on weekdays was significantly associated with lower percent body fat (PBF) and higher skeletal muscle mass index (SMMI), while sedentary behavior (SB) was correlated with lower soft lean mass index (SLMI), fat-free mass index (FFMI), and SMMI. Compositional isotemporal substitution analysis indicated that on weekdays, reallocating 5–30 minutes from SB to MVPA was associated with reduced PBF and higher muscle-related indices, whereas reallocating time from MVPA to SB resulted in adverse effects on these metrics, demonstrating an asymmetric pattern. No significant associations were observed between movement behaviors and body composition on weekends. The findings indicate that the association between MVPA and favorable body composition in preschoolers is significant on weekdays but not weekends, highlighting the context-dependent nature of movement behaviors in relation to body composition.

## INTRODUCTION

The preschool period represents a critical window for human growth and development, during which body composition, characterized by the relative amounts and distribution of adipose tissue, skeletal muscle, bone, and water, not only reflects a child’s current nutritional and activity status but also exerts a profound influence on long-term health trajectories. Studies have identified that obese preschoolers already exhibit early vascular alterations, including increased carotid artery intima-media thickness and elevated elastic modulus [1], these pathological changes may significantly elevate future cardiovascular disease risk. Concurrently, elevated C-reactive protein levels and other chronic inflammatory markers have been detected among overweight and obese preschool children, suggesting that a persistent low-grade inflammatory state may adversely affect health through multiple mechanisms [2, 3]. Furthermore, several studies have confirmed that insufficient skeletal muscle mass and strength in children and adolescents are associated with a markedly increased risk of metabolic dysfunction and cardiovascular diseases [4–6]. Therefore, maintaining appropriate body fat levels and adequate muscle mass is not only an important indicator of current nutritional status and growth in preschoolers but also a crucial factor for preventing metabolic disorders, reducing cardiovascular risk, and promoting lifelong health.

Physical activity (PA) has been widely recognized as a key behavioral determinant of body composition in children. Studies have reported positive associations between moderate-to-vigorous physical activity (MVPA) and both muscle mass and fat-free mass (FFM) in preschoolers [7, 8]. Regarding adiposity, one study found that each additional hour of MVPA was associated with a 0.8% reduction in percent body fat [9]. Research by Fu et al. also indicated that reallocating time to MVPA at the expense of other behaviors was negatively correlated with body fat percentage (PBF) in young children [10]. Similarly, Paul et al. observed an inverse relationship between MVPA and overall adiposity [11]. In contrast, Taylor et al. and Carson et al. reported no significant associations between movement behavior composition and adiposity indicators such as PBF [12, 13]. These inconsistencies may stem from variations in population characteristics or methodological approaches, or, potentially, the contextual settings in which physical activity occurs. A recent study revealed that structured MVPA was positively correlated with body shape score (calculated as height score × 0.2 + BMI score × 0.1) in preschoolers, whereas unstructured MVPA showed no such association [14]. This finding highlights the context-dependent effects of PA on body development across environmental contexts and underscores a significant gap in current research, specifically the oversight of how context-specific factors modulate the health effects of PA, an issue with considerable practical implications. Given the distinct behavioral patterns exhibited by preschoolers on weekdays (primarily kindergarten settings) versus weekends (primarily home settings), we hypothesize that movement behaviors may differentially influence body composition across these contexts.

Based on this background, the present study aims to use compositional data analysis to examine the context-specific associations between movement behaviors and body composition separately on weekdays and weekends. By comparing these context-dependent effects of movement behaviors in kindergarten-dominated versus homedominated settings and estimating the impact of time reallocation between movement behaviors on body composition, this research seeks to provide practical insights to inform educators and parents to optimize physical activity strategies for preschool children.

## MATERIALS AND METHODS

### Participants

This cross-sectional study was conducted from August to October 2022 in urban and suburban kindergartens in Beijing, China. A convenience sample of preschool children aged 3–6 years was recruited. The study protocol was approved by the Ethics Committee of the Capital Institute of Pediatrics (Approval No. SHERLL2021069), and written informed consent was obtained from all participating parents or guardians. Exclusion criteria included children with physical or intellectual disabilities; those with acute, chronic, or infectious diseases; and those whose motor function was impaired for any reason.

### Procedures

Prior to the commencement of the study, all participating teachers received comprehensive project briefing and training, which covered the research objectives, methodology, and overall procedures. It was emphasized that the study would not disrupt the children’s daily routines or activities. Following communication between the teachers and parents, informed consent was obtained from the parents, along with basic information about the children, including date of birth, gender, ethnicity, history of illnesses, and current health status.

Children who did not meet the inclusion criteria were excluded based on the study’s eligibility screening. Subsequently, accelerometers were distributed to parents for the objective monitoring of children’s movement behaviors throughout the measurement period. Training was conducted to instruct parents and teachers on the proper use of the devices and important precautions. Activity logs completed by parents and kindergarten teachers were used only to provide contextual information for interpreting accelerometer wear time (e.g., distinguishing between kindergarten and out-of-kindergarten periods) and identifying non-wear episodes (e.g., during sleep or bathing). To avoid potential interference between measurements, body composition assessments were conducted at the kindergarten after the accelerometers had been deployed.

### Measurements

#### Anthropometry

Anthropometric measurements were obtained following standardized procedures [15]. Height was measured using a Seca 217 stadiometer with a precision of 0.1 cm, and weight was assessed using a Seca 899 electronic scale with an accuracy of 0.1 kg. Children wore light clothing and stood barefoot during measurements. Each parameter was measured twice. If the difference between two height measurements exceeded 0.5 cm or between two weight measurements exceeded 0.25 kg, a third measurement was taken. The mean of the two closest measurements, provided they were within the acceptable discrepancy limits, was used as the final value. Waist circumference was measured twice at the midpoint between the lower edge of the rib cage and the upper edge of the iliac crest using a non-elastic tape; if the two measurements differed by less than 1 cm, the mean value was recorded. Body mass index (BMI) was calculated as weight in kilograms divided by the square of height in meters (kg/m^2^).

#### Movement behaviors monitoring

Following systematic training, accelerometers were distributed to parents with instructions for the child to wear the device during all waking hours for seven consecutive days, putting it on after waking in the morning and removing it before sleeping at night. The device was to be worn continuously except during water-based activities. Data were collected at a sampling rate of 30 Hz using 1-second epochs [15]. Non-wear time was identified using the Choi algorithm [16]. All analyses of physical activity duration, intensity, and patterning were based exclusively on these accelerometer data. Valid data were defined as wearing the device for at least 480 minutes per day, with a minimum of three valid weekdays and one valid weekend day required for inclusion. Physical activity intensity was classified into sedentary behavior (SB, ≤ 100 counts per minute, CPM), light physical activity (LPA, 101–1679 CPM), and moderateto-vigorous physical activity (MVPA, ≥ 1680 CPM) using standard count-per-minute thresholds previously validated in preschool populations [16]. Daily time spent in SB, LPA, and MVPA was calculated by summing the minutes of each intensity level and dividing by the number of valid days for each context (weekday/weekend). Sleep duration was obtained from parent-completed sleep logs, based on methods used in previous physical activity studies involving preschool children, including our earlier research [17].

#### Body composition

Body composition was assessed using the InBody J30 (Biospace, Korea), a tetra-polar bioelectrical impedance analysis (BIA) device. The validity of this method for use in preschool-aged children has been established, showing good agreement with dual-energy X-ray absorptiometry (DXA) [18]. Measurements included percent body fat (PBF), fat mass index (FMI), soft lean mass index (SLMI), fat-free mass index (FFMI), skeletal muscle mass index (SMMI), and bone mineral content index (BMCI). Prior to testing, children were instructed to remove any metal objects and stand barefoot on the device.

### Statistical analysis

All statistical analyses were performed using R version 4.1.3. Statistical significance was defined as *P* < 0.05 or a 95% confidence interval (95% CI) excluding zero. Compositional data analysis was conducted using the “compositions” and “robCompositions” packages. Continuous variables are presented as mean ± standard deviation, except for movement behavior data. Differences between genders were assessed using independent-samples t-tests for these variables. The central tendency and dispersion of 24-hour movement behavior data were described using geometric means and variation matrices, respectively. The geometric mean for each behavior was calculated and normalized to sum to 1440 minutes per day. In the compositional variation matrix, values closer to 0 indicate higher co-dependence between two behaviors, while values closer to 1 indicate greater independence. Differences in 24-hour movement behaviors between weekdays and weekends were examined using compositional multivariate analysis of variance (MANOVA).

Compositional multivariable linear regression models were employed to examine the associations between daily movement behaviors (explanatory variables) and each body composition indicator (dependent variable). Prior to inclusion in the models, each movement behavior was expressed as a set of three isometric log-ratio (ILR) coordinates. Four regression models were fitted for each body composition index to capture the aggregated relative influence of each movement behavior. Since the first ILR (ILR1) coordinate encapsulates all information regarding the composition of the four primary movement behaviors, only the gamma (γ) coefficient for ILR1 is reported to indicate the strength and direction of associations.

Following the methodology proposed by Dumuid et al., compositional isotemporal substitution analysis was used to estimate changes in body composition following time reallocation among movement behaviors [19]. Specifically, the aforementioned regression models were used to predict body composition at the baseline composition (i.e., mean time-use composition) and at new compositions resulting from time reallocations. The absolute change in body composition was derived by subtracting the predicted body composition value at the baseline time-use composition from the predicted value at the new composition. This procedure was repeated for pairwise time reallocations in 5-minute increments up to 30 minutes. To illustrate the doseresponse relationship, predicted changes in body composition were graphically presented across a continuum of time reallocations (0 to 30 minutes) involving moderate-to-vigorous physical activity (MVPA). All models were adjusted for age, sex, and body mass index (BMI).

## RESULTS

A total of 355 preschool children were initially enrolled in this study. After data screening, which excluded participants with missing sleep data or invalid accelerometer data, 268 children were included in the final analysis. Detailed information on participant characteristics, accelerometer wear parameters, and body composition measures, including fat, muscle, and bone mineral content index, is presented in Table S1. The analysis showed no significant differences between boys and girls in accelerometer wear time or in any body composition indicators.

The study identified significant differences in the time composition of movement behaviors between weekdays and weekends among preschool children. Specifically, time spent in moderate-to-vigorous physical activity (MVPA) and light physical activity (LPA) was significantly longer on weekends, while sleep duration was significantly longer on weekdays (Table 1). These findings suggest distinct movement behavior duration under different caregiving contexts. The compositional variation matrices for weekdays and weekends are presented in Table S2, respectively. Both matrices showed the greatest variance between sedentary behavior (SB) and MVPA, indicating the highest degree of independence between these two behaviors across both time periods.

**TABLE 1:** Geometric means for each movement behavior during time at weekdays and weekends

	Weekdays (min)	Weekends (min)	*P*
MVPA	**79.13 (5.50%)**	84.30** (5.85%)	
LPA	**110.90 (7.70%)**	114.32** (7.94%)	
SB	569.13 (39.52%)	570.17 (39.60%)	**0.001**
Sleep	680.84* (47.28%)	**671.20 (46.61%)**	
Total	1440 (100%)	1440 (100%)	

Note: Bold and denotes statistical significance. Abbreviations: MVPA, moderate-to-vigorous physical activity; LPA, light physical activity; SB, sedentary behavior.

** *P* < 0.01;

* *P* < 0.05.

Analysis of variance indicated that movement behaviors on weekdays were significantly associated with percent body fat (PBF), soft lean mass index (SLMI), fat-free mass index (FFMI), and skeletal muscle mass index (SMMI) (Table S3), whereas no significant associations were observed between weekend movement behaviors and any body composition indicators (Table S4). Further compositional multiple linear regression analysis revealed that increased time in MVPA on weekdays was associated with lower PBF and higher SMMI. Conversely, a greater proportion of sedentary behavior (SB) was negatively associated with all muscle-related indices, including SLMI, FFMI, and SMMI (Table 2). These results indicate that the observed associations between movement behaviors and body composition were specific to the weekday context. In contrast, no statistically significant relationship was identified within the weekend context.

**TABLE 2:** Multiple linear regression analyses of the association between daily movement behaviors and body composition on weekday and weekend

Dependent variable	Independent variable	Weekday		Weekend	

		γ-coefficient (95% *CI*)	*P*	γ-coefficient (95% *CI*)	P
**PBF(%)**	MVPA	**-6.1428 (-11.8946, -0.3910)**	**0.036**	-1.6523 (-5.9534, 2.6488)	0.450
	LPA	2.6692 (-5.3315, 10.6698)	0.512	0.6842 (-4.3452, 5.7135)	0.789
	SB	3.8043 (-3.9563, 11.5650)	0.335	1.5471 (-4.1565, 7.2507)	0.594
	Sleep	-0.3307 (-8.5195, 7.8582)	0.937	-0.5790 (-6.3686, 5.2106)	0.844

**FMI(kg/m^2^)**	MVPA	-0.6055 (-1.8646, 0.6536)	0.345	0.2386 (-0.6951, 1.1722)	0.615
	LPA	0.8853 (-0.8660, 2.6366)	0.320	-0.4352 (-1.5275, 0.6572)	0.434
	SB	-1.3762 (-3.0487, 0.2963)	0.106	0.1869 (-1.0549, 1.4286)	0.767
	Sleep	1.0964 (-0.6911, 2.8838)	0.228	0.0097 (-1.2507, 1.2702)	0.988

**SLMI(kg/m^2^)**	MVPA	0.8599 (-0.0652, 1.7850)	0.068	0.5153 (-0.1809, 1.2116)	0.146
	LPA	0.1180 (-1.1688, 1.4047)	0.857	-0.3558 (-1.1704, 0.4588)	0.391
	SB	**-1.5065 (-2.7353, -0.2776)**	**0.016**	-0.3335 (-1.2595, 0.5925)	0.479
	Sleep	0.5286 (0.1136, 2.0843)	0.429	0.1740 (-0.7660, 1.1139)	0.716

**FFMI(kg/m^2^)**	MVPA	0.9063 (-0.0758, 1.8884)	0.070	0.5195 (-0.2192, 1.2582)	0.167
	LPA	0.0519 (-1.3142, 1.4179)	0.940	-0.3850 (-1.2493, 0.4792)	0.381
	SB	**-1.5603 (-2.8649, -0.2557)**	**0.019**	-0.3231 (-1.3055, 0.6593)	0.518
	Sleep	0.6021 (-0.7921, 1.9963)	0.396	0.1887 (-0.8085, 1.1859)	0.710

**SMMI(kg/m^2^**)	MVPA	**0.7508 (0.1475, 1.3542)**	**0.015**	0.4176 (-0.0380, 0.8733)	0.072
	LPA	-0.1394 (-0.9786, 0.6700)	0.744	-0.3262 (-0.8593, 0.2069)	0.229
	SB	**-0.9560 (-1.7614, -0.1584)**	**0.019**	0.0871 (-0.5280, 0.7022)	0.781
	Sleep	0.3484 (-0.5081, 1.2050)	0.4239	-0.1785 (-0.7845, 0.4274)	0.562

**BMCI(kg/m^2^)**	MVPA	0.0465 (-0.0274, 0.1203)	0.217	0.0122 (-0.0427, 0.0670)	0.633
	LPA	-0.0519 (-0.1546, 0.0508)	0.321	-0.0311 (-0.0953, 0.0330)	0.340
	SB	-0.0634 (-0.1615, 0.0347)	0.204	-0.0014 (-0.0743, 0.0716)	0.970
	Sleep	0.0688 (-0.0360, 0.1737)	0.197	0.0204 (-0.0537, 0.0944)	0.588

Note: Analyses adjusted for age, gender, and BMI. Bold and denotes statistical significance. Abbreviations: MVPA, moderate-to-vigorous physical activity; LPA, light physical activity; SB, sedentary behaviour; *γ*-coefficient, gamma coefficients; ilr, isometric log ratio; CRF, cardiorespiratory fitness; PBF, percent body fat, FMI: fat mass index; SLMI, soft lean mass index; FFMI, fat-free mass index; SMMI, skeletal muscle mass index; BMCI, bone mineral content index; 95% CI, 95% confidence interval.

**FIG. 1:**
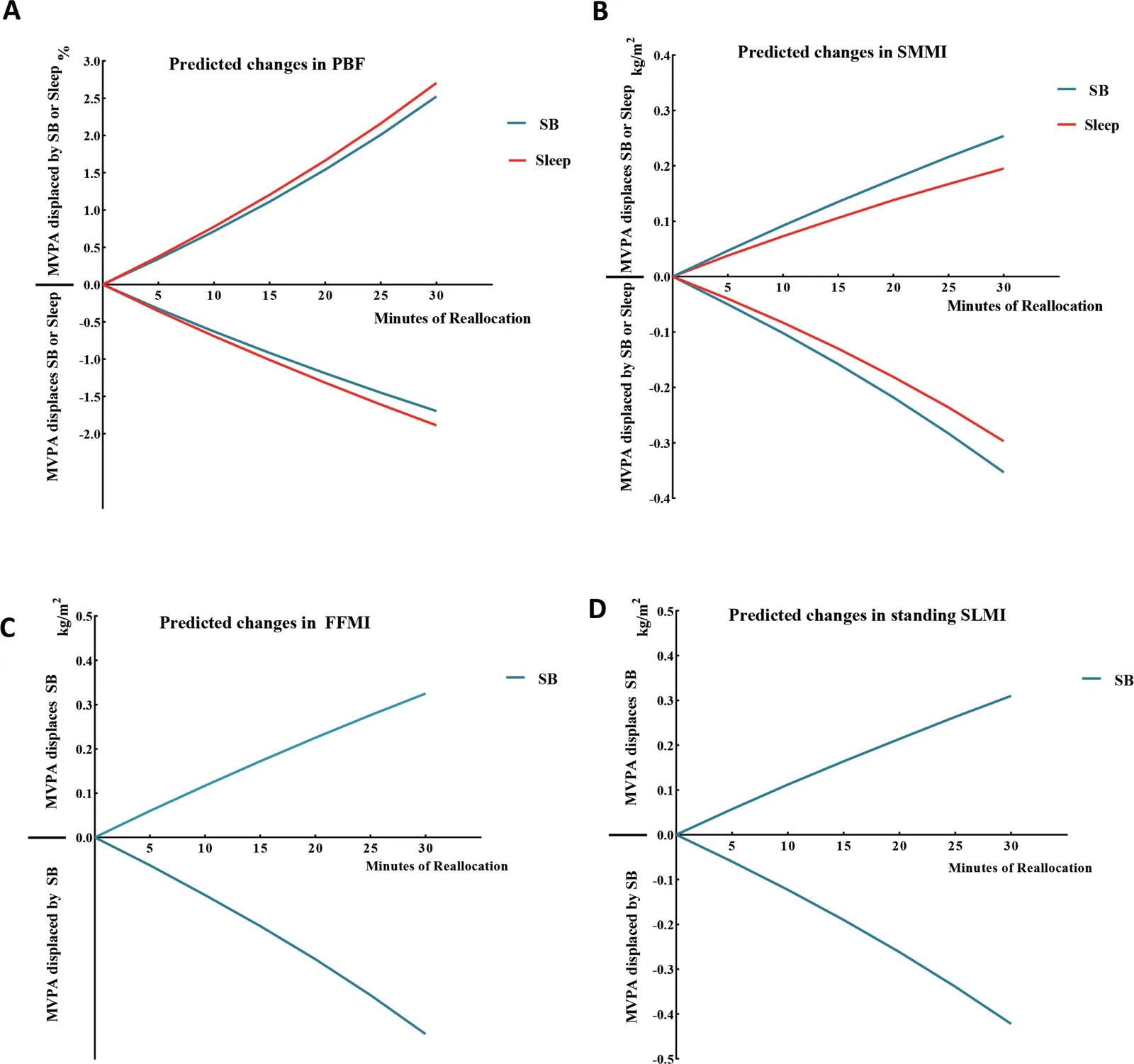
Asymmetry of predicted changes in body composition with the reallocation of time to and from MVPA time on weekdays. (A) Predicted changes in percent body fat (PBF). (B) Predicted changes in skeletal muscle mass index (SMMI). (C) Predicted changes in lean mass index (SLMI). (D) Predicted changes in fat-free mass index (FFMI). Line colors: The red line represents time reallocation between sleep and MVPA; the blue line represents time reallocation between sedentary behavior (SB) and MVPA. Directionality: In Panel A, the positive Y-axis indicates reallocation from MVPA to sleep or SB, and the negative Y-axis indicates reallocation from sleep or SB to MVPA; in Panels B, C, and D, the positive Y-axis indicates reallocation from SB or sleep to MVPA, and the negative Y-axis indicates reallocation from MVPA to SB or sleep. Asymmetry: The non-symmetric distribution of the lines about the Y-axis indicates that reallocating a fixed duration from MVPA to another behavior predicts a larger adverse change in the body composition metric than the beneficial change predicted by reallocating the same duration from that behavior to MVPA.

To further analyze the impact of reallocating time between movement behaviors on weekdays on body composition, compositional isotemporal substitution analysis was employed to examine the effects of time reallocation in durations ranging from 5 to 30 minutes. For all body composition parameters that exhibited a significant association with daily weekday movement behaviors, we performed compositional isotemporal substitution. As shown in Table 3, on weekdays, reallocating time from sedentary behavior (SB) to moderate-to-vigorous physical activity (MVPA) significantly decreased percent body fat (PBF) and significantly increased soft lean mass index (SLMI), fat-free mass index (FFMI), and skeletal muscle mass index (SMMI). Conversely, reallocating time from MVPA to SB resulted in a significant increase in PBF and a significant decrease in all muscle-related indices (SLMI, FFMI, SMMI). Notably, these associations exhibited an asymmetrical pattern: the beneficial effect of increasing MVPA (by reducing SB) on reducing PBF and enhancing muscle indices was smaller than the detrimental effect of reducing MVPA (by increasing SB) on these same metrics. Figure 1 visually summarizes the predicted changes in PBF, SLMI, FFMI, and SMMI based on isotemporal substitution, illustrating this asymmetry.

**TABLE 3:** Isotemporal substitutions between movement behaviors on weekday

Add	Remove	PBF (%)	SLMI (kg/m^2^)	FFMI (kg/m^2^)	SMMI (kg/m^2^)
**5 minutes reallocated**					
MVPA	LPA	-0.433 (-1.008, 0.142)	0.041 (-0.052, 0.134)	0.046 (-0.052, 0.144)	0.045 (-0.015, 0.106)
	SB	**-0.355 (-0.654, -0.056)**	**0.057 (0.009, 0.105**)	**0.060 (0.009, 0.111**)	**0.047 (0.016, 0.078**)
	Sleep	**-0.324 (-0.645, -0.003)**	0.042 (-0.009, 0.094)	0.044 (-0.011, 0.099)	**0.038 (0.004, 0.071**)

LPA	MVPA	0.449 (-0.131, 1.030)	-0.044 (-0.138, 0.049)	-0.049 (-0.148, 0.050)	-0.048 (-0.109, 0.013)
	SB	0.073 (-0.259, 0.405)	0.016 (-0.037, 0.069)	0.014 (-0.043, 0.070))	0.002 (-0.033, 0.037)
	Sleep	0.104 (-0.213, 0.421)	0.001 (-0.050, 0.052)	-0.002 (-0.056, 0.052)	-0.008 (-0.041, 0.026)

SB	MVPA	**0.376 (0.058, 0.694**)	**-0.060 (-0.111, -0.009**)	**-0.063 (-0.117, -0.009**)	**-0.050 (-0.083, -0.016**)
	LPA	-0.078 (-0.423, 0.268)	-0.016 (-0.072, 0.039)	-0.014 (-0.073, 0.045)	-0.002 (-0.038, 0.034)
	Sleep	0.031 (-0.073, 0.135)	-0.015 (-0.031, 0.002)	-0.016 (-0.033, 0.002)	-0.009 (-0.020, 0.001)

Sleep	MVPA	**0.345 (0.005, 0.686**)	-0.045 (-0.100, 0.010)	-0.047 (-0.106, 0.011)	**-0.040 (-0.076, -0.004**)
	LPA	-0.109 (-0.440, 0.223)	-0.001 (-0.055, 0.052)	0.002 (-0.055, 0.058)	0.008 (-0.027, 0.043)
	SB	-0.031 (-0.135, 0.073)	0.015 (-0.002, 0.031)	0.016 (-0.002, 0.033)	0.01 (-0.001, 0.020)

**10 minutes reallocated**					
MVPA	LPA	-0.852 (-2.000, 0.297)	0.079 (-0.106, 0.264)	0.089 (-0.107, 0.285)	0.089 (-0.032, 0.209)
	SB	**-0.692 (-1.271, -0.112**)	**0.112 (0.019, 0.205**)	**0.117 (0.019, 0.216**)	**0.092 (0.031, 0.153**)
	Sleep	**-0.629 (-1.253, -0.004**)	0.082 (-0.019, 0.182)	0.086 (-0.021, 0.192)	**0.073 (0.007, 0.138**)

LPA	MVPA	0.918 (-0.252, 2.088)	-0.092 (-0.280, 0.097)	-0.102 (-0.302, 0.098))	-0.098 (-0.221, 0.025)
	SB	0.141 (-0.510, 0.792)	0.032 (-0.073, 0.136)	0.028 (-0.083, 0.139)	0.004 (-0.064, 0.072)
	Sleep	0.204 (-0.418, 0.826)	0.002 (-0.098, 0.102)	-0.004 (-0.110, 0.102)	-0.015 (-0.080, 0.050)

SB	MVPA	**0.776 (0.117, 1.435**)	**-0.123 (-0.229, -0.018**)	**-0.130 (-0.242, -0.017**)	**-0.102 (-0.171, -0.033**)
	LPA	-0.161 (-0.867, 0.545)	-0.032 (-0.146, 0.081)	-0.028 (-0.148, 0.093)	-0.003 (-0.077, 0.071)
	Sleep	0.062 (-0.145, 0.269)	-0.029 (-0.062, 0.003)	-0.031 (-0.066, 0.004)	-0.019 (-0.040, 0.003)

Sleep	MVPA	**0.715 (0.011, 1.418)**	-0.094 (-0.207, 0.019)	-0.098 (-0.219, 0.022)	**-0.083 (-0.157, -0.010**)
	LPA	-0.223 (-0.900, 0.455)	-0.003 (-0.112, 0.106)	0.003 (-0.112, 0.119))	0.016 (-0.055, 0.087)
	SB	-0.063 (-0.270, 0.145)	0.030 (-0.003, 0.063)	0.032 (-0.004, 0.067)	0.019 (-0.002, 0.041)

**15 minutes reallocated**					
MVPA	LPA	-1.259 (-2.982, 0.464)	0.114 (-0.163, 0.392)	0.130 (-0.165, 0.424)	0.130 (-0.051, 0.311)
	SB	**-1.011 (-1.857, -0.166**)	**0.164 (0.028, 0.300**)	**0.172 (0.028, 0.316**)	**0.135 (0.047, 0.224**)
	Sleep	**-0.917 (-1.830, -0.004**)	0.119 (-0.028, 0.266)	0.125 (-0.031, 0.281)	**0.106 (0.010, 0.202**)

LPA	MVPA	1.411 (-0.362, 3.185)	-0.144 (-0.429, 0.142)	-0.159 (-0.462, 0.144)	-0.152 (-0.338, 0.034)
	SB	0.205 (-0.754, 1.164)	0.048 (-0.106, 0.202)	0.042 (-0.122, 0.205)	0.007 (-0.093, 0.107)
	Sleep	0.300 (-0.616, 1.215)	0.003 (-0.145, 0.150)	-0.006 (-0.162, 0.151)	-0.022 (-0.118, 0.074)

SB	MVPA	**1.204 (0.179, 2.229**)	**-0.190 (-0.355, -0.026**)	**-0.200 (-0.375, -0.025**)	**-0.158 (-0.266, -0.051**)
	LPA	-0.250 (-1.333, 0.832)	-0.049 (-0.223, 0.125)	-0.042 (-0.226, 0.143)	-0.004 (-0.117, 0.109)
	Sleep	0.092 (-0.218, 0.403)	-0.044 (-0.094, 0.005)	-0.047 (-0.099, 0.006)	-0.028 (-0.061, 0.004)

Sleep	MVPA	**1.112 (0.020, 2.204**)	-0.147 (-0.322, 0.029)	-0.154 (-0.340, 0.033)	**-0.130 (-0.245, -0.015**)
	LPA	-0.342 (-1.383, 0.699)	-0.005 (-0.172, 0.163)	0.005 (-0.173, 0.183)	0.024 (-0.085, 0.133)
	SB	-0.094 (-0.406. 0.217)	0.045 (-0.005, 0.094)	0.047 (-0.005, 0.100)	0.029 (-0.004, 0.061)

**20 minutes reallocated**					
MVPA	LPA	-1.659 (-3.961, 0.644)	0.148 (-0.223, 0.518)	0.168 (-0.226, 0.562)	0.171 (-0.071, 0.412)
	SB	**-1.317 (-2.415, -0.219**)	**0.214 (0.038, 0.391**)	**0.225 (0.038, 0.412**)	**0.176 (0.061, 0.291**)
	Sleep	**-1.190 (-2.378, -0.003**)	0.154 (-0.037, 0.345)	0.161 (-0.042, 0.364)	**0.138 (0.013, 0.262**)

LPA	MVPA	1.933 (-0.462, 4.329)	-0.200 (-0.586, 0.186)	-0.221 (-0.631, 0.188)	-0.209 (-0.461, 0.042)
	SB	0.265 (-0.991, 1.522)	0.064 (-0.138, 0.265)	0.056 (-0.158, 0.270)	0.010 (-0.122, 0.141)
	Sleep	0.392 (-0.806, 1.590)	0.003 (-0.190, 0.196)	-0.008 (-0.213, 0.197)	-0.029 (-0.155, 0.097)

SB	MVPA	**1.664 (-0.242, 3.085**)	**-0.262 (-0.490, -0.034**)	**-0.275 (-0.518, -0.033**)	**-0.218 (-0.367, -0.069**)
	LPA	-0.346 (-1.824, 1.132)	-0.065 (-0.303, 0.172)	-0.056 (-0.308, 0.196)	-0.005 (-0.160, 0.150)
	Sleep	0.122 (-0.291, 0.536)	-0.059 (-0.125, 0.007)	-0.062 (-0.132, 0.008)	-0.038 (-0.081, 0.005)

Sleep	MVPA	**1.542 (0.031, 3.052**)	-0.204 (-0.447, 0.039)	-0.214 (-0.472, 0.044)	**-0.181 (-0.339, -0.022**)
	LPA	-0.468 (-1.890, 0.955)	-0.007 (-0.236, 0.222)	0.006 (-0.237, 0.249)	0.033 (-0.117, 0.182)
	SB	-0.126 (-0.542, 0.290)	0.060 (-0.006, 0.126)	0.063 (-0.007, 0.134)	0.038 (-0.005, 0.085)

**25 minutes reallocated**					
MVPA	LPA	-2.051 (-4.942, 0.840)	0.178 (-0.287, 0.644)	0.204 (-0.290, 0.698)	0.209 (-0.094, 0.513)
	SB	**-1.609 (-2.946, -0.271**)	**0.263 (0.049, 0.478**)	**0.276 (0.048, 0.504**)	**0.216 (0.076, 0.356**)
	Sleep	**-1.450 (-2.900, -0.0001**)	0.187 (-0.046, 0.421)	0.196 (-0.052, 0.444)	**0.167 (0.015, 0.319**)

LPA	MVPA	2.490 (-0.552, 5.532)	-0.262 (-0.752, 0.228)	-0.289 (-0.809, 0.231)	-0.271 (-0.591, 0.048)
	SB	0.322 (-1.222, 1.866)	0.079 (-0.168, 0.327)	0.070 (-0.193, 0.333)	0.013 (-0.149, 0.174)
	Sleep	0.481 (-0.990, 1.952)	0.004 (-0.233, 0.240)	-0.010 (-0.262, 0.241)	-0.036 (-0.190, 0.119)

SB	MVPA	**2.162 (0.309, 4.015**)	**-0.339 (-0.636, -0.041**)	**-0.356 (-0.672, -0.040**)	**-0.283 (-0.477, -0.089**)
	LPA	-0.449 (-2.343, 1.445)	-0.082 (-0.386, 0.222)	-0.070 (-0.392, 0.253)	-0.005 (-0.203, 0.193)
	Sleep	0.152 (-0.365, 0.669)	-0.073 (-0.156, 0.009)	-0.078 (-0.165, 0.010)	-0.047 (-0.101, 0.007)

Sleep	MVPA	**2.010 (0.046, 3.974**)	-0.266 (-0.582, 0.050)	-0.279 (-0.615, 0.056)	**-0.236 (-0.442, -0.030**)
	LPA	-0.601 (-2.426, 1.224)	-0.010 (-0.303, 0.284)	0.007 (-0.304, 0.319)	0.042 (-0.150, 0.233)
	SB	-0.158 (-0.679, 0.362)	0.075 (-0.008, 0.158)	0.080 (-0.008, 0.167)	0.048 (-0.006, 0.102)

**30 minutes reallocated**					
MVPA	LPA	-2.439 (-5.931, 1.053)	0.207 (-0.355, 0.769)	0.238 (-0.359, 0.835)	0.247 (-0.120, 0.614)
	SB	**-1.889 (-3.455, -0.322**)	**0.310 (0.059, 0.561**)	**0.325 (0.059, 0.592**)	**0.254 (0.090, 0.418**)
	Sleep	-1.697 (-3.398, 0.004)	0.219 (-0.055, 0.493)	0.229 (-0.062, 0.519)	**0.195 (0.017, 0.374**)

LPA	MVPA	3.089 (-0.633, 6.811)	-0.331 (-0.930, 0.269)	-0.363 (-1.000, 0.273)	-0.339 (-0.730, 0.052)
	SB	0.375 (-1.448, 2.198)	0.095 (-0.197, 0.388)	0.084 (-0.227, 0.394)	0.016 (-0.175, 0.207)
	Sleep	0.566 (-1.168, 2.301)	0.004 (-0.275, 0.283)	-0.013 (-0.309, 0.284)	-0.042 (-0.225, 0.140)

SB	MVPA	**2.705 (0.378, 5.032**)	**-0.422 (-0.796, -0.048**)	**-0.444 (-0.840, -0.047**)	**-0.353 (-0.596, -0.109**)
	LPA	-0.560 (-2.893, 1.773)	-0.099 (-0.474, 0.275)	-0.084 (-0.481, 0.314)	-0.005 (-0.249, 0.240)
	Sleep	0.182 (-0.438, 0.802)	-0.088 (-0.186, 0.011)	-0.093 (-0.198, 0.012)	-0.056 (-0.121, 0.008)

Sleep	MVPA	**2.523 (0.063, 4.984**)	-0.335 (-0.731, 0.061)	-0.352 (-0.772, 0.069)	**-0.297 (-0.555, -0.039**)
	LPA	-0.741 (-2.992, 1.509)	-0.012 (-0.375, 0.350)	0.008 (-0.376, 0.393)	0.051 (-0.185, 0.287)
	SB	-0.191 (-0.816, 0.434)	0.090 (-0.009, 0.190)	0.096 (-0.010, 0.201)	0.058 (-0.007, 0.123)

Note: Analyses adjusted for age, gender, and BMI. Bold and denotes statistical significance. Abbreviations: MVPA, moderate-to-vigorous physical activity; LPA, light physical activity; SB, sedentary behaviour; *γ*-coefficient, gamma coefficients; ilr, isometric log ratio; CRF, cardiorespiratory fitness; PBF, percent body fat, FMI: fat mass index; SLMI, soft lean mass index; FFMI, fat-free mass index; SMMI, skeletal muscle mass index; BMCI, bone mineral content index; 95% CI, 95% confidence interval.

Regarding sleep reallocation, reallocating 5 to 30 minutes of sleep to MVPA resulted in a significant increase in skeletal muscle mass index (SMMI), also demonstrating an asymmetric pattern. For percent body fat (PBF), displacing 5 to 25 minutes of sleep with MVPA was associated with a reduction in PBF; however, reallocating 30 minutes of sleep to MVPA did not yield a significant effect. Sleep may thus be a relevant, though less consistent, factor for body composition in preschoolers compared to MVPA and sedentary behavior, warranting further study. It is also noteworthy that sleep quality depends not only on duration but also on factors such as sleep architecture and the proportion of deep sleep, with both excessively long and short durations being potentially detrimental to health. The variability observed in sleep reallocation effects across different time intervals, both in magnitude and direction, could partially explain its lower analytical stability relative to the more consistent patterns seen for MVPA and sedentary behavior.

## DISCUSSION

To our knowledge, this is the first study to separately analyze and compare the associations between 24-hour movement behaviors and body composition on weekdays versus weekends in preschoolaged children. Using compositional data analysis, the present study revealed that on weekdays, moderate-to-vigorous physical activity (MVPA) was significantly negatively associated with percent body fat (PBF) and positively associated with skeletal muscle mass index (SMMI), while sedentary behavior (SB) showed significant negative associations with muscle-related indices, including soft lean mass index (SLMI), fat-free mass index (FFMI), and SMMI. Further isotemporal substitution analysis indicated that reallocating time from SB to MVPA was associated with reduced PBF and increased SLMI, FFMI, and SMMI. In contrast, reallocating time from MVPA to SB was linked to increased PBF and impaired muscle development, with these effects exhibiting a distinct asymmetrical pattern. However, no significant associations were observed between movement behaviors and body composition metrics on weekends. These findings highlight the significant association between weekday movement behaviors and healthier body composition in preschool children, while also emphasizing how environmental differences shape the context-dependent relationship between movement behaviors and body composition in this population.

The present study found that moderate-to-vigorous physical activity (MVPA) on weekdays was negatively associated with percent body fat (PBF). This result is consistent with previous studies that did not differentiate between weekdays and weekends. A recent compositional data analysis conducted in Chinese children aged 3–6 years also reported a negative association between MVPA and body fat percentage (BFP) [10]. Furthermore, a study of 4-year-old Swedish children similarly indicated that higher levels of MVPA were associated with lower fat mass percentage [8]. Another cohort study in preschoolers further demonstrated that daily MVPA accumulated in bouts lasting at least 1 minute was significantly associated with a 1.3% reduction in body fat percentage [9]. However, some studies have failed to observe a significant association between physical activity and adiposity indicators [7, 20]. Additionally, Paul et al. reported an inverse relationship between MVPA and overall adiposity, noting that this association was primarily driven by vigorous physical activity (VPA) [11]. We suggest that the absence of a significant association between movement behaviors and adiposity parameters during weekends in the current study may be attributable to contextual differences in the quality of MVPA. Specifically, higher-quality MVPA, which may include a greater proportion of VPA, is more characteristic of structured weekday environments and could explain the divergent relationships with body composition across different settings.

The study also revealed differential associations between MVPA and various adiposity indicators: MVPA showed a significant negative correlation with percent body fat (PBF) but no statistically significant association with fat mass index (FMI). This discrepancy may be explained by the dual effect of MVPA, which can simultaneously promote muscle development and suppress fat accumulation. Another potential factor is the distinct body composition characteristics of preschool children, in whom FMI remains stable with increasing age [21]. Consequently, in this population, PBF may be a more sensitive indicator than FMI for detecting qualitative changes in body composition attributable to MVPA.

The results of this study indicate that moderate-to-vigorous physical activity (MVPA) on weekdays was positively associated with skeletal muscle mass index (SMMI), while sedentary behavior (SB) was negatively associated with soft lean mass index (SLMI), fat-free mass index (FFMI), and SMMI. These correlations are consistent with previous research. A cross-sectional study involving children aged 5 to 6 years reported a significant correlation between muscle mass and MVPA [22], and another cross-sectional study in 4-year-olds found that higher-intensity physical activity was associated with greater fat-free mass index [7]. Intervention studies have further demonstrated the beneficial effects of MVPA in increasing both skeletal muscle mass (SMM) and fat-free mass (FFM) [23]. Moreover, the current results align with muscle ultrasonography studies: Pengyu et al. observed that total physical activity and MVPA were positively correlated with muscle thickness in the anterior tibial and posterior lower leg muscles in preschoolers [24], and another study by the same group identified a significant positive association between weekday MVPA and posterior thigh muscle thickness [25].

It is noteworthy that the current study observed inconsistencies in the associations between movement behaviors and muscle-related indicators. Specifically, MVPA was significantly associated only with SMMI, but not with SLMI or FFMI. This discrepancy may be attributed to differences in the physiological composition of these indices. SMMI specifically reflects skeletal muscle, which is most directly responsive to physical activity. SLMI includes metabolically less active tissues such as visceral organs, and FFMI is further influenced by components such as bone minerals. Therefore, it is plausible that skeletal muscle mass is more sensitive to stimuli induced by physical movement. At the mechanistic level, MVPA may promote angiogenesis and muscle tissue repair by elevating levels of nitric oxide (NO) and vascular endothelial growth factor A (VEGF-A) [26]. Furthermore, MVPA may stimulate muscle protein synthesis through activation of the mTOR signaling pathway and modulation of ribosomal biogenesis and epigenetic mechanisms [27]. Collectively, these data suggest that increasing weekday MVPA by decreasing SB represents a modifiable determinant of musculoskeletal development in preschool-aged children.

This study found that higher levels of moderate-to-vigorous physical activity (MVPA) on weekdays were significantly associated with lower percent body fat and higher skeletal muscle mass index. However, although MVPA duration was longer on weekends, no significant associations were observed with body composition indicators during this period. This observed weekday-weekend discrepancy should be interpreted with caution. We suggest that the health effects of physical activity in preschoolers are context-dependent, extending beyond mere duration to encompass qualitative differences in movement behavior. This pattern may not solely reflect a true effect specific to weekdays. Alternative explanations must be considered, including possible methodological differences in measurement context, sample characteristics, or random variation. Therefore, we propose that the observed difference is best understood not as an effect of the weekday itself, but as reflecting systematic differences in activity patterns and qualitative dimensions between the structured environment of kindergarten days and the less-regulated context of weekends. As noted by Delong et al., only structured MVPA was significantly positively associated with body shape indicators in preschool children [14]. In the current study, children spent most of their waking hours on weekdays in kindergarten settings, where physical activity largely consisted of structured activities with clear goals and rules. Examples include group games and organized running and jumping tasks, which commonly incorporated competitive elements to achieve objectives, alongside peer interaction and imitation mechanisms. This combination of structure and social engagement tends to elicit higher intensity levels, thereby providing stronger stimulation to metabolic function and the musculoskeletal system, ultimately leading to more substantial health benefits [28]. In contrast, weekend activities were largely unstructured, driven primarily by children’s spontaneous interests rather than systematic health-promoting design, such as unstructured chasing games. These activities are also susceptible to external factors such as parental physical activity levels and accessibility of community sports facilities, which may limit the realization of health benefits from weekend MVPA [29].

Consequently, despite longer total movement time on weekends, the lower average intensity and more sporadic distribution of activity in this context may weaken its detectable association with body composition. This pattern implies that increasing MVPA from a relatively constrained baseline may yield more pronounced compositional benefits than comparable increases from an already elevated activity level. These observations point toward a potential “optimal benefit pattern” in physical activity, shaped by the interplay of duration, intensity, and temporal distribution, and likely moderated by individual factors such as developmental stage, baseline fitness, and genetic predispositions, warranting further investigation.

Furthermore, several studies have reported that MVPA levels on weekends are actually lower than on weekdays among preschool children. For example, a UK study showed that the proportion of time spent in MVPA was 6.3% on weekdays compared to 2.0% on weekends [30]. Similarly, a Swedish study found that the time spent in kindergarten during weekdays was the most active period of the day for 4-year-olds, while the same time window on weekends was the least active [31]. This relative deficiency in weekend activity volume may further attenuate any potential positive effects on body composition.

Collectively, these findings highlight the significant and contextspecific influence of weekday structured activity on body composition. The structured, high-intensity, and socially interactive activity patterns typically provided in kindergarten settings may elicit stronger physiological stimulation compared to the fragmented and spontaneous unstructured activities common on weekends. Therefore, in addition to activity duration, the quality of physical activity during weekends may be an important consideration for future interventions.

Therefore, future research and practical efforts should consider not only the quantity but also the context and quality of physical activity, particularly in understanding how to support meaningful movement across different daily settings such as kindergarten and home environments. By optimizing activity programs, they can systematically increase children’s engagement in MVPA and reduce sedentary behavior during kindergarten hours, thereby effectively supporting healthy body composition development. This approach is particularly important for children with obesity, who often exhibit low duration of unstructured physical activity, a pattern that may perpetuate a negative cycle. High-quality structured activities can serve as a critical means to disrupt this cycle. Meanwhile, during weekends, when care is primarily provided at home, caregivers could actively encourage children to participate in regular and goal-oriented play activities. Such initiatives may help compensate for the limited health benefits associated with unstructured activities and comprehensively promote healthy growth in preschool children.

We acknowledge several limitations in this study that should be considered. First, the cross-sectional nature of this study limits the ability to establish causal relationships between physical activity and body composition. The direction of these associations and their underlying mechanisms warrant further investigation through longitudinal or intervention studies. Second, the use of a convenience sampling method may limit the representativeness of the sample and introduce selection bias, thereby affecting the generalizability of the findings. Third, sleep data were collected via parent-reported questionnaires rather than objective measures, which may introduce recall bias or reporting errors and compromise the accuracy of sleep duration and quality assessment. Fourth, the multiple regression models conducted across different movement components and outcomes were not adjusted for multiple comparisons, which should be considered when interpreting the results. Additionally, potential confounding factors, such as nutritional intake, genetic background, and family socioeconomic status were not systematically controlled, each of which may significantly influence body composition in children. Finally, the observed context-dependent associations may also be influenced by methodological and confounding factors. Consequently, future research should adopt more consistent measurement protocols between weekdays and weekends, incorporate objective sleep monitoring instruments, apply appropriate statistical corrections for multiple comparisons, systematically account for nutritional and socioeconomic factors, and employ more rigorous sampling strategies. Such integrated approaches would help to more comprehensively and accurately elucidate the complex relationship between physical activity and body composition in preschoolers.

## CONCLUSIONS

In this study, we employed compositional data analysis to investigate weekday and weekend movement behaviors in preschoolers. The analysis revealed that moderate-to-vigorous physical activity (MVPA) on weekdays was associated with lower percent body fat (PBF) and higher skeletal muscle mass index (SMMI), while sedentary behavior (SB) was negatively associated with soft lean mass index (SLMI), fat-free mass index (FFMI), and SMMI. Isotemporal substitution analysis indicated that on weekdays, reallocating time from SB to MVPA improved body composition, whereas the reverse substitution produced adverse effects, demonstrating an asymmetric pattern. No significant association was observed between weekend activity and body composition. These findings underscore a distinct context-dependent pattern, wherein significant associations between movement behaviors and body composition were evident during weekdays but not during weekends. This context encompasses organizational features such as the activity structure, the nature of socio-emotional interactions during play, and the regularity of daily schedules. These contextual elements collectively influence key characteristics of physical activity, including its total volume, intensity distribution, and intermittency patterns, which may modulate its physiological stimulus and subsequent health benefits.

Consequently, future research and practical initiatives should account for this context-dependency. A promising direction involves developing tailored strategies for each setting: in kindergartens, enhancing the intensity and intermittency of structured play; at home, supporting families in creating more regular and patterned opportunities for vigorous activity during weekends. Unraveling the specific determinants of this context-dependent relationship will be crucial for designing effective interventions to promote healthy physical development across all facets of a child’s life.

## Supplementary Material

Weekday-specific associations of movement behaviors with body composition in preschool children: A compositional data analysis
