# Supplementary material for: Weekday-specific associations of movement behaviors with body composition in preschool children: A compositional data analysis
Source: Biol Sport. 2026 Apr 20;43:1315–27. doi: 10.5114/biolsport.2026.159563 (PMC13504950; doi:10.5114/biolsport.2026.159563)
Supplement: Supplementary file 1 [file JBS-43-57602-s1.pdf]

SUPPLEMENTARY MATERIAL

TABLE S1. The descriptive characteristics, accelerometer-measured time, and body composition parameters of the participants

	Overall (n =268)	Boys (n =145)	Girls (*n* =123)	*P*
**Participant characteristics**				
Age (y)	4.98 ± 0.77	5.07 ± 0.72	4.87 ± 0.81	**0.04**
Height (cm)	110.54 ± 7.07	111.38 ± 7.24	109.56 ± 6.77	**0.04**
Weight (kg)	19.13 ± 3.57	19.52 ± 3.88	18.67 ± 3.11	**< 0.05**
BMI (kg/m^2)	15.55 ± 1.63	15.61 ± 1.73	15.48 ± 1.51	0.54
WHtR(%)	46.78 ± 3.68	46.83 ± 3.52	46.71 ± 3.86	0.80
**Accelerometer-measured time**				
Valid days on Weekdays (d),	4.44 ± 0.91	4.45 ± 0.90	4.44 ± 0.92	0.93
Wearing on Weekdays (min/d),	669.09 ± 53.45	665.19 ± 50.07	673.69 ± 57.04	0.20
Valid days on Weekends (d),	1.97 ± 0.58	1.97 ± 0.55	1.98 ± 0.63	0.80
Wearing on Weekends (min/d),	714.46 ± 87.98	718.36 ± 82.87	709.87 ± 93.79	0.43
**Body Composition Variables**				
PBF(%)	19.29 ± 8.68	19.22 ± 9.14	19.37 ± 8.15	0.89
FMI(kg/m^2)	3.06 ± 1.60	3.06 ± 1.69	3.06 ± 1.49	0.98
SLMI(kg/m^2)	11.78 ± 1.21	11.83 ± 1.31	11.71 ± 1.08	0.39
FFMI(kg/m^2)	12.43 ± 1.29	12.49 ± 1.40	12.37 ± 1.15	0.45
SMMI(kg/m^2)	5.71 ± 0.84	5.77 ± 0.93	5.64 ± 0.72	0.65
BMCI(kg/m^2)	0.66 ± 0.10	0.66 ± 0.11	0.66 ± 0.09	0.59

Note: Bold and denotes statistical significance
Abbreviations: BMI, body mass index; WHtR, waist to height ratio; PBF, percent body fat, FMI: fat mass index; SLMI, soft lean mass index; FFMI, fat-free mass index; SMMI, skeletal muscle mass index; BMCI, bone mineral content index;

TABLE S2. Compositional variation matrix for movement behaviors on weekdays

	movement behaviors	Sleep	SB	LPA	MVPA
weekdays	Sleep	0.000	0.035	0.078	0.139
	SB	0.035	0.000	0.084	0.152
	LPA	0.078	0.084	0.000	0.063
	MVPA	0.139	0.152	0.063	0.000
weekends	Sleep	0.000	0.021	0.034	0.071
	SB	0.021	0.000	0.037	0.088
	LPA	0.034	0.037	0.000	0.040
	MVPA	0.071	0.088	0.040	0.000

Abbreviations: MVPA, moderate-to-vigorous physical activity; LPA, light physical activity; SB, sedentary behaviour

TABLE S3. The contribution of movement behaviors on weekday to the explanation of variance in each measure of body composition variables

Dependent variable	Sum sq.	*df*	*F* – value	*p* – value
PBF(%)	370.690	3	2.324	**0.075**
FMI(kg/m^2)	7.642	3	0.997	0.395
SLMI(kg/m^2)	18.233	3	4.408	**< 0.01**
FFMI(kg/m^2)	19.378	3	4.157	**< 0.01**
SMMI(kg/m^2)	9.414	3	5.350	**< 0.01**
BMCI(kg/m^2)	0.040	3	1.504	0.214

Note: Analyses adjusted for age, gender, and BMI. Bold and denotes statistical significance ($P < 0.1$). Abbreviations: df, degrees of freedom; PBF, percent body fat, FMI: fat mass index; SLMI, soft lean mass index; FFMI, fat-free mass index; SMMI, skeletal muscle mass index; BMCI, bone mineral content index;

TABLE S4. The contribution of movement behaviors on weekend to the explanation of variance in each measure of body composition variables

Dependent variable	Sum sq.	*df*	*F* – value	*p* – value
PBF(%)	61.035	3	0.374	0.772
FMI(kg/m^2)	1.782	3	0.231	0.875
SLMI(kg/m^2)	4.080	3	0.949	0.417
FFMI(kg/m^2)	3.977	3	0.822	0.483
SMMI(kg/m^2)	2.305	3	1.252	0.291
BMCI(kg/m^2)	0.012	3	0.451	0.717

Note: Analyses adjusted for age, gender, and BMI. Bold and denotes statistical significance ($P < 0.1$). Abbreviations: df, degrees of freedom; PBF, percent body fat, FMI: fat mass index; SLMI, soft lean mass index; FFMI, fat-free mass index; SMMI, skeletal muscle mass index; BMCI, bone mineral content index;
